# Bibliometric analysis of stroke and quality of life

**DOI:** 10.3389/fneur.2023.1143713

**Published:** 2023-04-11

**Authors:** Mengtong Chen, Yuting Zhang, Lijuan Dong, Xiaomei Guo

**Affiliations:** ^1^Department of Nursing, Zhongshan Hospital of Traditional Chinese Medicine, Zhongshan, Guangdong, China; ^2^Clinical Medical College of Acupuncture, Moxibustion, and Rehabilitation, Guangzhou University of Chinese Medicine, Guangzhou, Guangdong, China

**Keywords:** stroke, quality of life, bibliometric analysis, CiteSpace, VOSviewer

## Abstract

**Objective:**

To perform a bibliometric analysis of stroke and quality of life studies between 2000 and 2022 using VOSviewer and CiteSpace.

**Methods:**

The literature data source for this study was the Web of Science Core Collection. CiteSpace and VOSviewer were used to analyze publications in relation to authors, countries, institutions, journals, references, and keywords.

**Results:**

A total of 704 publications were obtained for the bibliometric analysis. The number of publications has gradually increased over 23 years, with an annual increase of 728.6%. Kim S is the most prolific author in the field (10 publications), and the United States and Chinese University of Hong Kong have the most publications. Stroke is the most prolific journal with the most citations per paper (91.58) and the highest impact factor (IF 2021, 10.17). The most high-frequency keywords are “stroke,” “quality of life,” “rehabilitation,” and “depression.”

**Conclusion:**

A bibliometric analysis of stroke and quality of life over the last 23  years provides future research directions.

## 1. Introduction

Stroke is an acute cerebrovascular disease caused by the sudden rupture of a blood vessel in the brain or the blockage of a blood vessel that prevents blood from flowing to the brain. It is a group of diseases that cause brain tissue damage, including ischemic and hemorrhagic strokes. The Global Burden of Disease Study 2017 reported that China had approximately 2 million deaths due to stroke in 2017 (1, 2) and that stroke has become the second leading cause of death worldwide (3). The American Heart Association and the American Stroke Association predict that the total annual cost of stroke expenditure will reach $24,067 million by 2030 (4), imposing a huge global social and economic burden. After a stroke occurs, approximately half of the survivors have physical, psychological, and social impairments. These impairments will lead to increased dependency on activities of daily living, mood changes, and social distancing (5), which have a significant impsignificantly impact patients’ quality of survival of life (QOL) refers to a person’s sense of well-being, life goals, autonomy, ability to assume valuable roles, and the ability to participate in important relationships (6) and covers physical health, material health, social health, emotional health, development, and activity (7). Standardized scale rating methods are commonly used to evaluate QOL, and commonly used scales include the EuroQol Five Dimensions Questionnaire (EQ-5D) (8), Barthel Index (BI) (9), Health Status Survey Questionnaire SF36 (10), Stroke Specific Quality of Life Scale (SS-QOL) (11), Hamilton Anxiety Scale (HAMA) (12), Hamilton Depression Scale (HAMD) (12). Functional status, depression, social support, age, gender and race were important predictors of quality of life, among which functional status and depression were the most important determinants (5).

Traditional reviews emphasize the content of the articles and do not reveal the topical issues and collaborations. Bibliometric analysis is a mathematical and statistical method that uses software to extract literature data such as authors, countries, institutions, keywords, cited authors, and cited journals to reveal the macroscopic pattern of a field through quantitative analysis (13), which can effectively explore the development of a subiect (14). Bibliometrics has a wide range of applications, including Biomedicine, Humanities and Social Sciences, Engineering and Technology, Economic Management, etc. (15). Current bibliometric analysis software includes CiteSpace (16) (Drexel University, Philadelphia, PA, the United States) and VOSviewer (17) (Leiden University, Leiden, Netherlands), through software-generated collaborative networks and co-occurrence networks, etc., we can identify potential collaborators, and the evolution of thematic research hotspots provides scholars with a dynamic development process in the field. It is useful for scholars to have a comprehensive understanding of the current state of research in the field (18) and to predict the future research hotpots. The literature related to stroke and quality of life has a tendency to increase year by year, but a bibliometric analysis of the topic of stroke and quality of survival has not been conducted; therefore, a bibliometric analysis of the topic is necessary to help researchers understand the hot spots and emerging trends in the field of stroke and quality of life.

## 2. Materials and methods

### 2.1. Search strategy

The Web of Science Core Collection (WoSCC) was used as the source of bibliographic data. Web of Science has been widely accepted by researchers as a high-quality digital literature resource database and is generally considered the most suitable database for bibliometric analysis, and the citation index was chosen as Science Citation Index Expanded (SCI-EXPANDED) - 1900-present in order to ensure comprehensive and accurate search data. Search strategy: TI = (stroke* OR “Cerebrovascular Accident*” OR CVA* OR “Cerebrovascular Apoplexy” OR “Brain Vascular Accident*” OR “Cerebrovascular Stroke*” OR Apoplexy OR “Cerebral Stroke*” OR “Acute Stroke*” OR “Acute Cerebrovascular Accident*”) and TI = (“quality of life” OR “QOL” OR “Life Quality” OR “Health-Related Quality Of Life” OR “Health Related Quality Of Life” OR “HRQOL”). The search period was limited to January 1, 2000, to November 1, 2022, with Article and Review selected as the document type and English as the language. 704 articles were obtained after de-duplication.

### 2.2. Analytical tools

**Figure 1:**
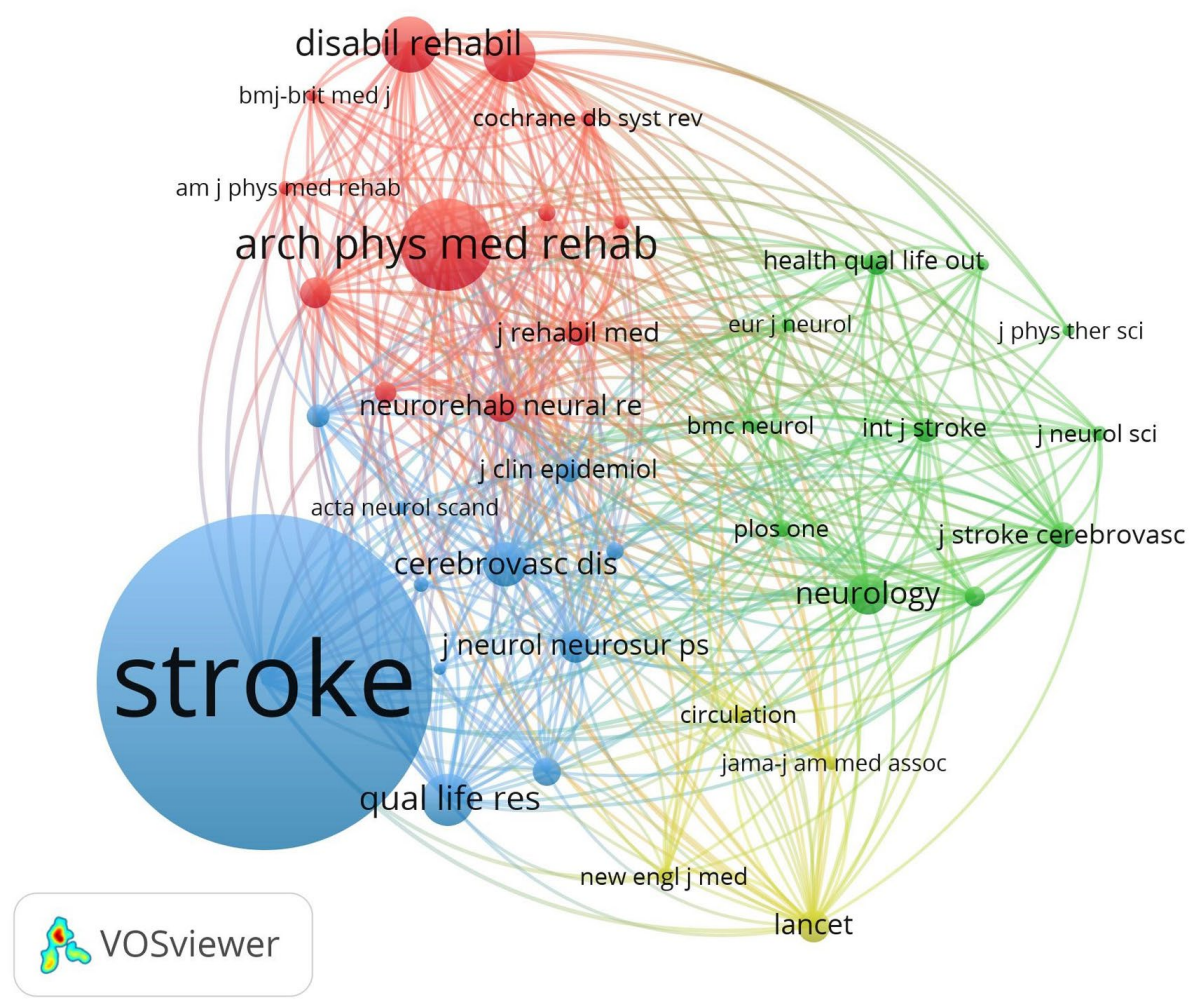
Map of co-cited journals with publications.

**Figure 2:**
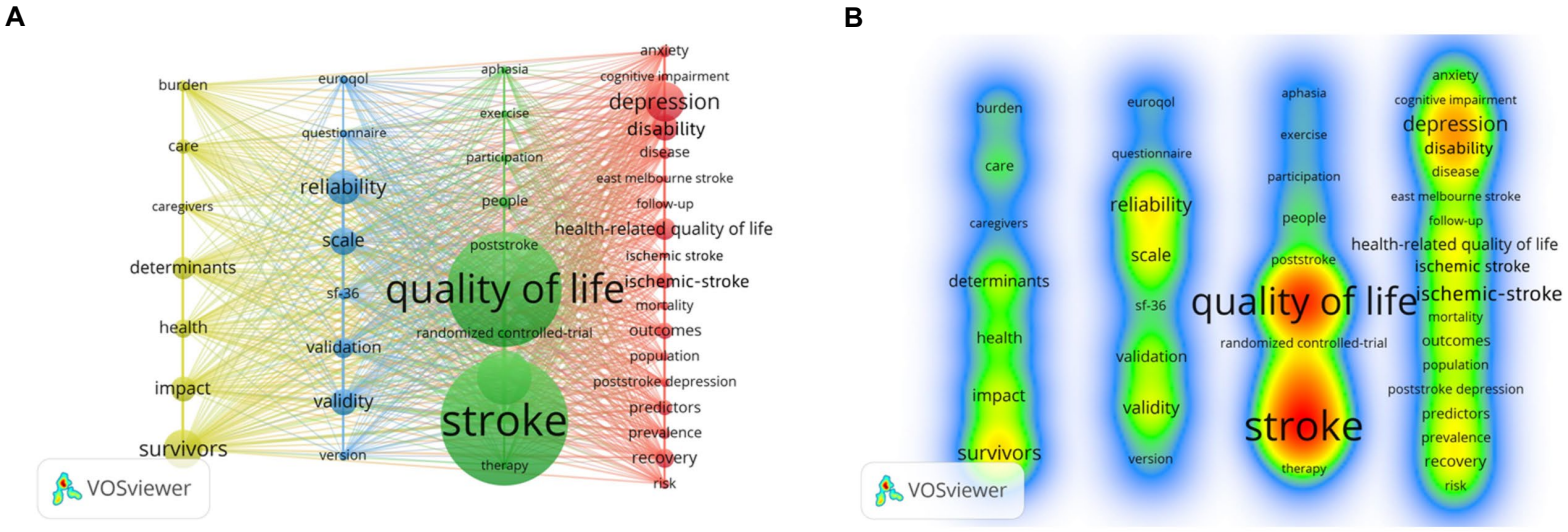
**(A)** Map of co-occurring keywords with occurrences. **(B)** Map of keywords density.

Microsoft Excel 2019 was used to produce annual, and annual cumulative publication volume and citation co-citation count graphs for analyzing the global output and trends of papers related to stroke and quality of survival. CiteSpace 6.1.R3 and VOS Viewer 1.6.17 were used to produce network graphs to extract and analyze the number of publications (including output, authors, journals, countries, and institutions), citation frequency (including co-cited authors, co-cited journals, and co-cited literature), and co-occurring keywords to track research trends and hotspots. Both software is information visualization software based on the Java platform. CiteSpace is used to generate collaborative networks (including authors, countries, and institutions), co-citation networks (co-cited authors), and co-occurrence networks (strongest burst references, keywords). In CiteSpace network mapping, the larger the node, the higher the number of articles represented or the higher the frequency of co-occurrence, and the nodes between VOS Viewer is used to generate co-occurrence network (keywords), co-citation network (cited journals, co-cited literature). VOS Viewer generates the clustering view (Figure 1) and density view (Figure 2B) respectively In the cluster view, the color of the node represents the cluster it belongs to, the size of the node represents the frequency of co-occurrence, the line between the nodes represents the co-occurrence (or co-citation) relationship, and the thicker or thinner of them indicates the co-occurrence (or co-citation) intensity. In the density view, each node on the plot is filled with color according to the density of the elements around that node, the higher the density, the closer to red; on the contrary, the lower the density, the closer to blue.

### 2.3. Research ethics

The study was conducted as a bibliometric analysis. All data sources were available on the Internet, and no animal or human subjects were involved. Therefore, permission was not required from the ethics committee.

## 3. Results

### 3.1. Analysis of publications outputs and citations

**Figure 3:**
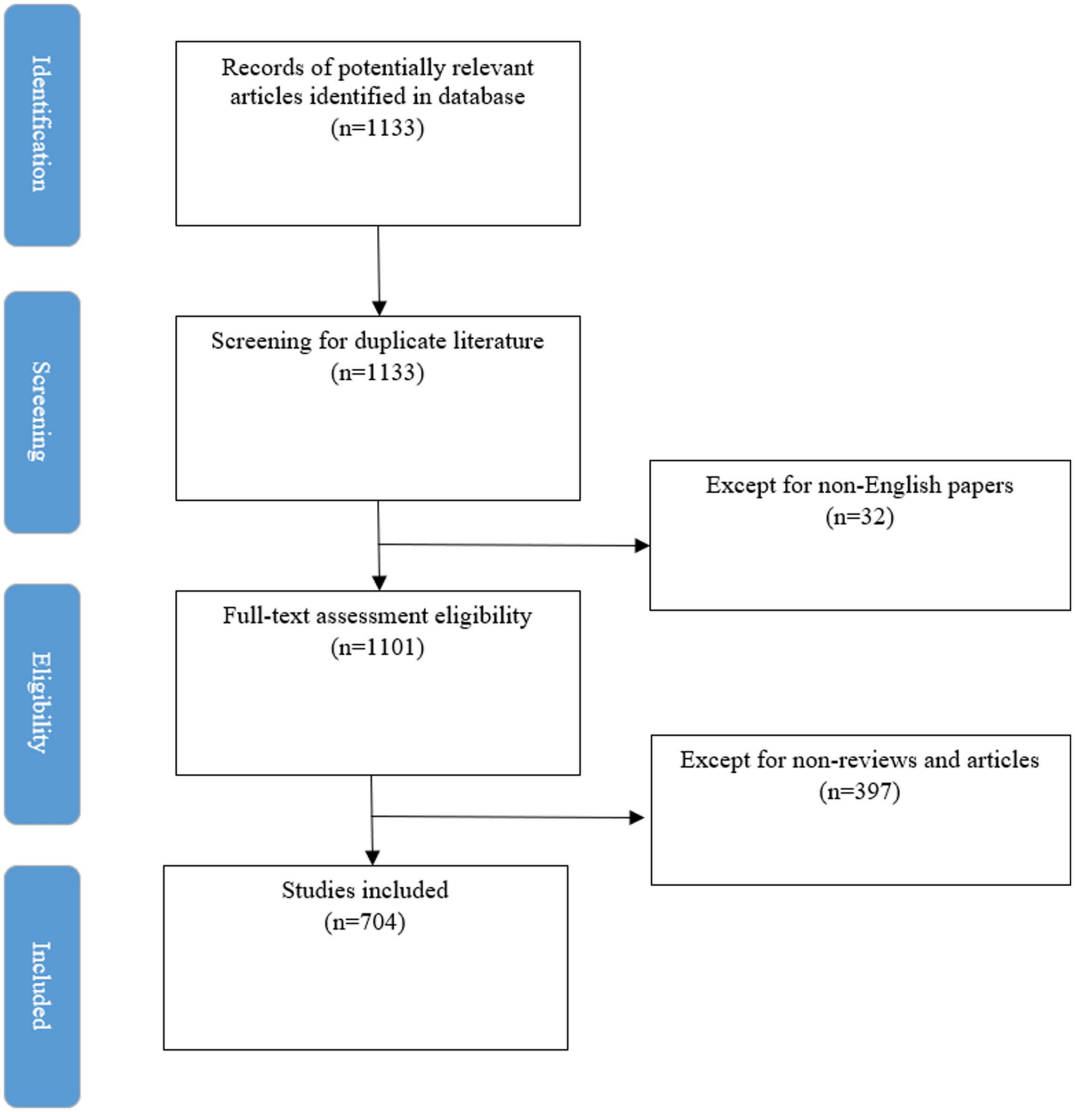
The flow chart of screening process.

**Figure 4:**
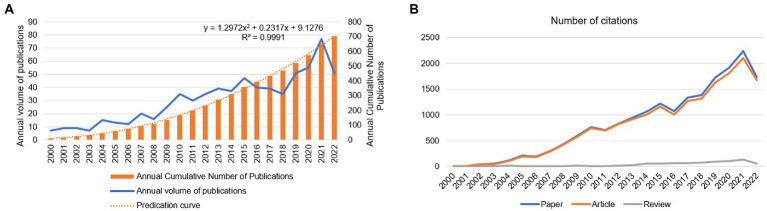
The number of publications and citations. **(A)** The number of publication outputs and growth trend from 2000 to 2022. **(B)** The number of annual citations from 2000 to 2022.

Between January 1, 2000, and November 1, 2022, the Web of Science Core Collection included 704 articles that met the inclusion criteria, including 668 articles and 36 reviews, with an average annual yield of 31 articles (Figure 3). We obtained graphs of the number of publications and citations (Figure 4) using Microsoft Excel. Overall, there is a steady growth trend in the number of articles published and frequently cited, with an annual increase of 728.6% (from 7 articles in 2000 to 51 articles in 2022). We fitted a polynomial to the cumulative annual number of publications (Figure 4A) with a growth trend model of *R*^2^ = 0.9991, predicting that more articles on quality of life after stroke will be published in the future. Figure 4B shows that 704 papers were cited 18,922 times (H-index 68), with an average citation per paper of 26.88 and an average annual citation frequency of 823, with 2,236 citations in 2021, the highest ever, and 1734 citations in the first 10 months of 2022. 1,677 citations in 2022, which is 29.42 times that of the overview.

### 3.2. Analysis of authors and co-cited authors

**Figure 5:**
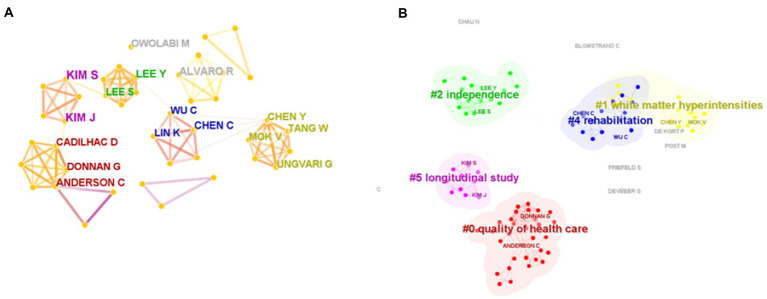
**(A)** Map of authors with publications. **(B)** Map of clustering of authors.

Author collaboration mapping was generated by CiteSpace (Figure 5A), and a total of 3,261 authors were involved in 704 publications. The size of nodes represents their publication volume, and Table 1 lists the top five authors in terms of publication volume, with Kim S ranking first (10 publications), followed by Kim J (9 publications), and Anderson C (8 publications) in third place. The connecting lines between nodes represent the existence of collaborative relationships among authors, and we performed a cluster analysis of the author collaboration network using keywords to extract cluster labels, forming a total of five key clusters (Figure 5B). By calculating the centrality, we found that the highest centrality in author collaboration was 0.03, and centrality greater than 0.1 was considered an important node, which indicates that the connection between authors is not strong and the collaboration between authors should be strengthened in the future.

**Table 1:** Top five active authors and co-cited authors.

Rank	Author	Count	Co-cited author	Cited times	Co-cited author	Centrality
1	Kim S	10	Williams LS	158	Ali M	0.21
2	Kim J	9	Duncan PW	144	Anderson C	0.2
3	Anderson C	8	Ware JE	138	Ahlsio B	0.14
4	Chen C	8	Carod-artal FJ	117	Dorman PJ	0.13
5	Alvaro R	8	Sturm JW	114	Brott T	0.12

**Figure 6:**
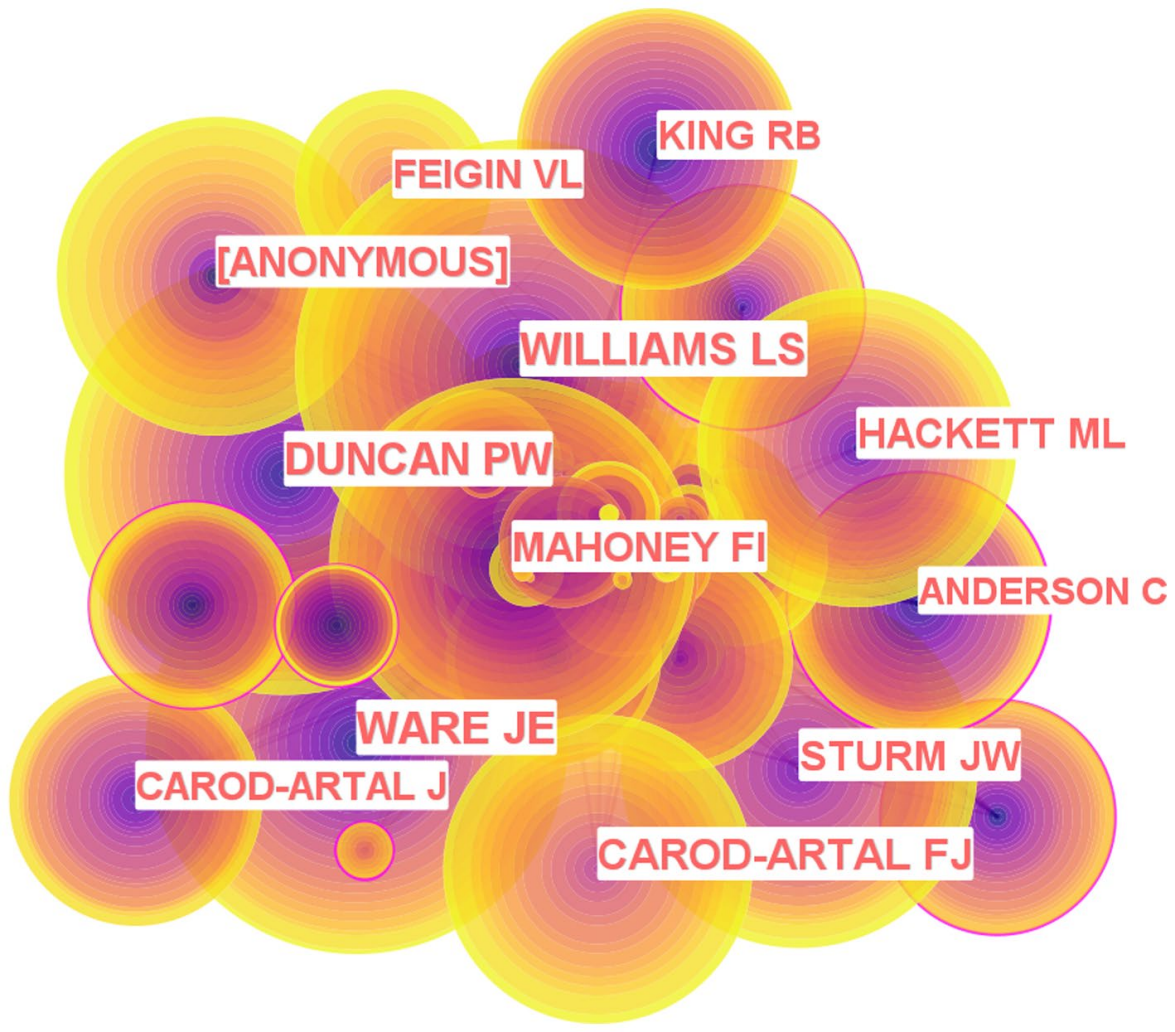
Map of co-cited authors with publications.

The network diagram of co-cited authors is shown in Figure 6. Among all co-cited authors, Williams LS from Indiana University School of Medicine ranked first with 158 cited times, followed by Duncan PW, Ware JE, with 144 and 138 cited times, respectively, and the fourth and fifth co-cited authors were Carod-artal FJ (117 cited times) and Sturm JW (114 cited times). Among all co-cited authors, Ali M was the first with a high centrality of 0.21, followed by Anderson C (0.2), Ahlsio B (0.14), Dorman PJ (0.13), and Brott T (0.12), all of whom are influential researchers in the field.

### 3.3. Analysis of countries and institutions

**Figure 7:**
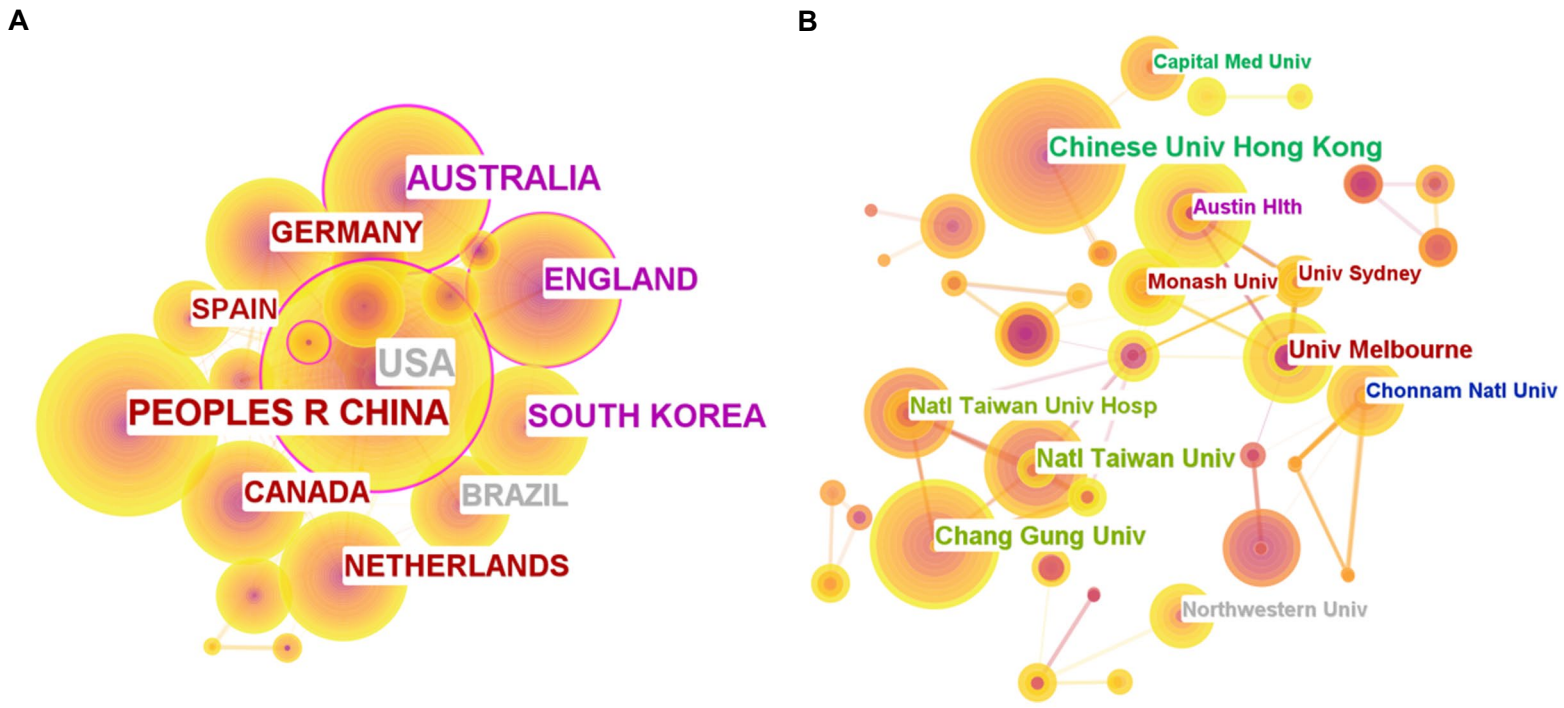
**(A)** Map of countries. **(B)** Map of institutions.

**Figure 8:**
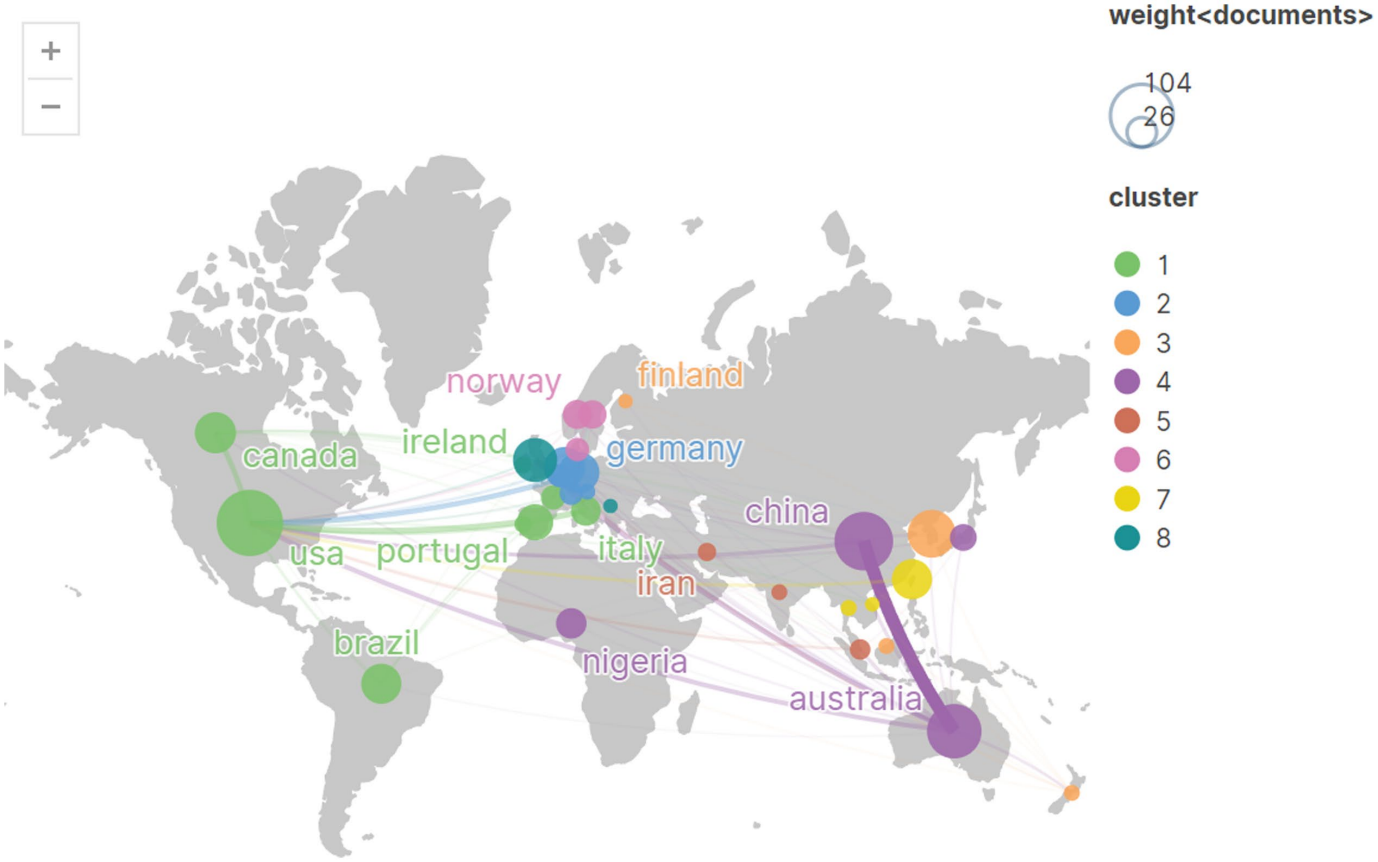
The distribution of countries/region.

An analysis of the country collaboration network through CiteSpace (Figure 7A) showed that a total of 66 countries or regions contributed to publications related to stroke and quality of life, and the top five countries in terms of number of publications were the United States, China, Australia, South Korea, and the United Kingdom, with the exception of China, which is a developing country, and the remaining four countries are all developed countries, which shows that developed countries play an important role in this research field. The purple circle represents the centrality, and the center of the study is the most important one. The purple circle represents centrality, and centrality greater than 0.1 is considered an important node. The United States, Australia, the United Kingdom, Nigeria, and Singapore are the top five countries in terms of centrality and have an important impact on global scientific research cooperation. The countries or regions contributing in this area are shown in the world map in Figure 8.

Figure 7B shows a map of institutional collaboration networks in each country and region. Four of the top five institutions in terms of number of publications are from China and one from Australia, with Chinese University of Hong Kong ranking first (20 publications), Chang Gung University ranking second (15 publications), and National Taiwan University ranking third (15 publications). Using CiteSpace to calculate the mediated centrality of research institutions, we found that three of the top five countries for centrality were from Australia, with Deakin University ranking first with a mediated centrality of 0.06. As an international comprehensive university, it is one of the youngest but most dynamic universities in Australia, in addition to the Deakin University Language Centre being one of the best language schools in Australia. The third-ranked university, King’s College London, is from the UK and the fifth-ranked university, Chonnam National University, is from South Korea. The centrality of the research institutions is all less than 0.1, indicating that inter-institutional links are not strong and inter-institutional cooperation should be strengthened in the future.

### 3.4. Distribution of journals and co-cited journals

**Figure 9:**
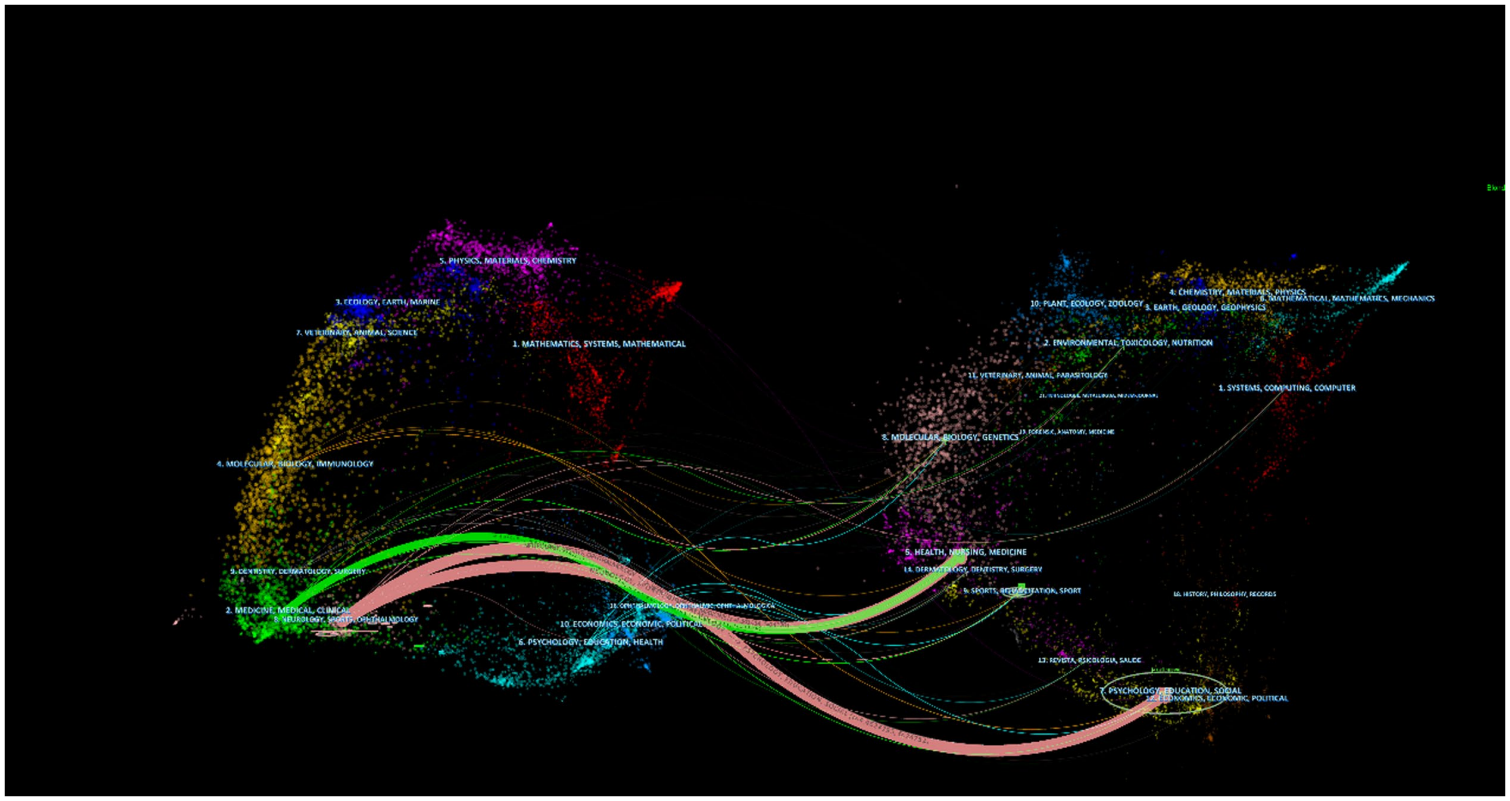
The dual-map overlay of journals with publications.

A total of 222 journals have published papers on the quality of life and stroke. Figure 9 shows a dual- map of journals. The left is the citation map, the right is the cited map, and the curves are citation linkages. The linkage trajectories provide an interdisciplinary understanding of the field, and the *z*-Scores function highlights the more fluid trajectories, with higher scores indicated by thicker linkages. In this case, publications in the neurology, sports, ophthalmology (pink trajectory) domain are clearly influenced by publications in the psychology, education, social (*z* = 4.46, *f* = 24,732) and health, nursing, medicine (*z* = 4.34, *f* = 24,120) domains. In addition, publications in the medicine, medical, clinical (green trajectory) domains were influenced by publications in the health, nursing, medicine (*z* = 1.89, *f* = 11,736) domain. Table 2 lists the top 10 academic journals that published papers related to the quality of survival after stroke research. Sorted by publications, Stroke tops the list with 53 articles and plays a significant role in the field. It was followed by Topics in stroke rehabilitation (28 articles) and Disability and rehabilitation (27 articles). The average impact factor of the top 10 journals was 3.709, with the highest impact factor being the most prolific stroke (IF = 10.17). The vast majority of journals are based in the United Kingdom, with all journals based in developed countries.

**Table 2:** Top 10 academic journals based on publications.

Rank	Source (Abbreviations)	Publications	Citations	Average Citations/Publications	Country	IF (2021)
1	Stroke	53	4,854	91.58	United States	10.17
2	Topics in stroke rehabilitation (top stroke rehabil)	28	566	20.21	United Kingdom	2.177
3	Disability and rehabilitation (disabil rehabil)	27	554	20.52	United Kingdom	2.439
4	Journal of stroke and cerebrovascular diseases (j stroke cerebrovasc)	25	301	12.04	United Kingdom	2.677
5	Quality of life research (qual life res)	25	768	30.72	Netherlands	3.44
6	Archives of physical medicine and rehabilitation (arch phys med rehab)	24	1,216	50.67	United Kingdom	4.06
7	Health and quality of life outcomes (health qual life out)	22	486	22.09	United Kingdom	3.077
8	Cerebrovascular diseases (cerebrovasc dis)	18	1,045	58.06	Switzerland	3.104
9	Neurorehabilitation	15	110	7.33	Netherlands	1.986
10	Journal of rehabilitation medicine (j rehabil med)	12	384	32.00	United Kingdom	3.959

For further analysis of journal co-citations, we used VOSviewer software to perform a citation co-citation analysis of 38 journals with a threshold of 100 citations, which resulted in Figure 1, with four clusters corresponding to the four colors in the figure. The red clusters are mainly journals in the field of rehabilitation medicine, focusing on the application of rehabilitation techniques and comprehensive care tools in stroke disease; The green and blue clusters are mainly journals in neurology and healthy quality of life assessment, which tend to have more research on the prevention, diagnosis and management of stroke-related diseases and the assessment of medical therapies and quality of life, and are dedicated to reducing the global burden of stroke; The yellow clusters are mainly comprehensive journals of clinical medicine, which contain many relevant clinical and laboratory trials that provide information on how to improve patient prognosis. A review of articles from these journals can provide theoretical and empirical support for this study. Table 3 and Figure 1 show that the most cited is Stroke (4131), followed by Archives of physical medicine and rehabilitation (980), and Disability and rehabilitation (579). Centrality was calculated by CiteSpace software and the journal with the highest centrality is Physical therapy (0.07) as seen in Table 3.

**Table 3:** Top five co-cited journals in terms of counts and centrality.

Rank	Co-cited counts	Cited journal (Abbreviations)	Centrality	Cited journal (Abbreviations)
1	4,131	Stroke	0.07	Physical therapy (phys ther)
2	980	Archives of physical medicine and rehabilitation (arch phys med rehab)	0.06	International journal of rehabilitation research (int j rehabil res)
3	579	Disability and rehabilitation (disabil rehabil)	0.06	American journal of physical medicine & rehabilitation (am j phys med rehab)
4	529	Clinical rehabilitation (clin rehabil)	0.06	Acta psychiatrica scandinavica (acta psychiat scand)
5	525	Quality of life research (qual life res)	0.05	Journal of the neurological sciences (j neurol sci)

### 3.5. Analysis of co-cited references

**Figure 10:**
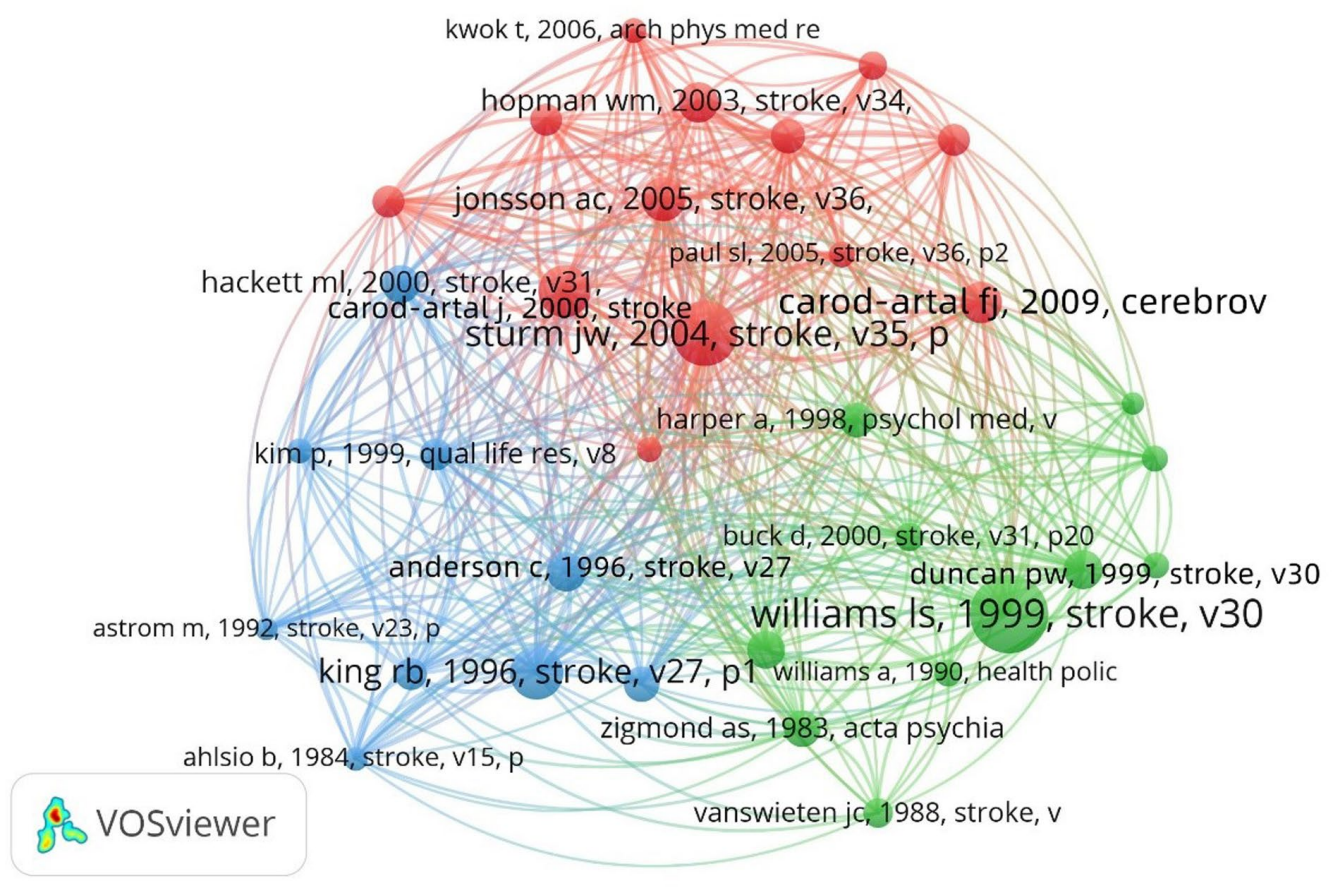
Map of co-cited references with citations.

To analyze the co-citation of the literature, we used VOSviewer to plot the total number of references as 15,318, set the minimum number of co-citations of the literature as 35, and screened 33 papers for co-citation analysis of the cited literature, and constructed a network within the field of stroke and quality of life research (Figure 10).

Table 4 lists the top 10 most frequently cited articles, involving 9 clinical studies, 1 review, and highly cited literature mostly published from 1996 to 2005. The most frequently cited are Williams et al. (11) (120 times), Sturm et al. (19) (100 times). The literature for the green clusters in Figure 10 focuses on the assessment of the characteristics of HRQOL-related scales in stroke patients. The most common generic scale for assessing HRQOL in stroke patients is the SF-36 (20), which has a floor effect in terms of physical functioning, but it is limited in that it does not reflect well the level of social functioning of patients and tends to trigger a ceiling effect (19). Therefore, it should be supplemented with specific stroke HRQOL scales, such as SS-QOL (11) and SIS (21) scales, which have better reliability, validity and responsiveness. In addition, we should use a patient-centered approach to participate in scale production (22) with robust psychometric and quality-of-life measures, and patients should be involved in each stage of scale development. The red and blue clustered literature focuses on how overall and domain-specific HRQOL is assessed in stroke survivors with caregivers at different times and the main predictors of quality of life after stroke. The independent determinant of HRQOL in stroke survivors is functional status, which is low, resulting in social isolation and reduced social activity with little social support, making a depression in stroke survivors frequent (23). The quality of life of stroke survivors is highly correlated with demographic variables, such as gender, age, and racial. Women, especially older women, who have survived after stroke have lower levels of all measures of quality of life and much higher rates of depression than men. This is because in many countries, women take on the responsibility for household management and have greater difficulty maintaining this role after stroke. Multiple studies using data from the Reasons for Geographic and Racial Differences in Stroke (REGARDS) study have found that blacks have lower rates of blood pressure control than whites with or without stroke (24). Racial differences in the incidence of cardiovascular events and quality of life of stroke survivors are largely due to differences in healthy lifestyles across groups (25). We considered the top 10 references based on the number of citations as valuable references. These references are landmark references in the field and set the stage for future research.

**Table 4:** Top 10 co-cited references in terms of citations.

Rank	Title	citations	year	First author	Journal	Document Type
1	Development of a Stroke-Specific Quality of Life Scale	120	1999	Williams LS	Stroke	Article
2	Quality of Life After Stroke: The North East Melbourne Stroke Incidence Study (NEMESIS)	100	2004	Sturm JW	Stroke	Article
3	Quality of Life After Stroke	82	1996	King RB	Stroke	Article
4	Quality of Life Among Stroke Survivors Evaluated 1 Year After Stroke: Experience of a Stroke Unit	80	2000	Carod-artal J	Stroke	Article
5	Determinants of Quality of Life in Stroke Survivors and Their Informal Caregivers	68	2005	Jonsson AC	Stroke	Article
6	Quality of Life after Stroke: The Importance of a Good Recovery	63	2009	Carod-artal FJ	Cerebrovascular Diseases	review
7	Quality of Life During and After Inpatient Stroke Rehabilitation	62	2003	Hopman WM	Stroke	Article
8	Validation of the Short Form 36 (SF-36) Health Survey Questionnaire Among Stroke Patients	61	1996	Anderson C	Stroke	Article
9	The Stroke Impact Scale Version 2.0. Evaluation of Reliability, Validity, and Sensitivity to Change	61	1999	Duncan PW	Stroke	Article
10	Health-Related Quality of Life Among Long-Term Survivors of Stroke: Results From the Auckland Stroke Study, 1991–1992	59	2000	Hackett ML	Stroke	Article

### 3.6. Analysis of co-occurring keywords

The keyword co-occurrence map reflects the core themes and research hotspots in the field of quality of life after stroke research. As of November 2022, a total of 2016 keywords have appeared in the field of stroke and quality of life. In the VOSview software, we set the frequency of displayed co-occurrence to at least 25 times. Thus, a total of 43 focused keywords were included (Figure 2A). We used Pajek software to arrange the keywords in columns according to different clusters, with nodes indicating keywords; the larger the node area, the more frequently the keyword appears, and the lines between the nodes indicate the strength of association. Figure 2B shows the density view, the density size depends on the number and importance of keywords, and this figure is used to quickly observe the knowledge and the research density of the domain. We found four different colored clusters, with red clusters representing studies of stroke disease progression and related complications, green clusters representing studies of clinical treatments for stroke and changes in functional outcomes after stroke, blue clusters representing studies of standardized quality-of-life assessment measures and measurement tools after stroke, and yellow representing studies of factors related to the level of health care and the magnitude of caregiver burden for stroke patients (Table 5). To get a clearer picture of the specifics of the keywords, the high-frequency keyword assemblies with a co-occurrence frequency of more than 80 are presented in Table 6, and as seen in Figure 2B and Table 6, “stroke” (451 times), “quality of life” (403 times), “rehabilitation “(194 times), “depression” (140 times), and “survivors” (138 times) constitute the representative terms and hot topics in this field.

**Table 5:** Clusters of co-occurring keywords in areas.

Cluster	Color	Keywords
1	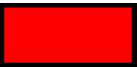	Anxiety; cognitive impairment; depression; disability; disease; east Melbourne stroke; follow-up; health-related quality; ischemic stroke; ischemic-stroke; mortality; outcomes; population; poststroke depression; predictors; prevalence; recovery; risk.
2	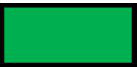	Aphasia; exercise; participation; people; poststroke; quality of life; randomized controlled-trial; stroke; therapy.
3	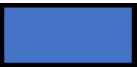	Questionnaire; reliability; scale; sf-36; validation; validity; version; euroqol.
4	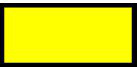	Burden; care; caregivers; determinants; health; survivors.

**Table 6:** Top 10 keywords.

Rank	Occurrences	Keyword	Rank	Occurrences	Keyword
1	451	Stroke	6	122	Reliability
2	403	Quality of life	7	98	Scale
3	193	Rehabilitation	8	93	Validity
4	140	Depression	9	93	Disability
5	138	Survivors	10	88	Impact

Depression refers primarily to post-stroke depression, which is a treatable condition, and early intervention can prevent its progression to chronic depression, thereby slowing the recovery process. In addition to stroke survivors, the psychological status of family caregivers should also be taken into account, and studies have shown that the prevalence of depression in caregivers is twice as high as that in older adults in the community (26). The important predictor of depression is social support (27). The isolation and reduced social activities caused by physical disability in stroke survivors predispose to depression, while the patient’s functional status, level of dependency, and family and social support can determine the quality of life of the caregiver (23). Stroke survivors will receive intensive rehabilitation in the hospital and will be less motivated to rehabilitate after discharge from the hospital, when it is more important to enhance home rehabilitation, including promoting low to moderate intensity physical activity, muscle strengthening exercises and reducing sedentary behavior, and changing poor sleep habits (short <6 h or long >8 h of sleep) (28). Caregivers are trained by professionals during patient rehabilitation, and group exercises and group activities are provided in the community to promote socialization and reintegration of stroke patients into community life (29). In addition, it is necessary to strengthen mental health management for stroke survivors and family caregivers by providing interventions such as support groups and counseling.

## 4. Discussion

The field of health quality of life research for stroke survivors is a multidisciplinary research area that combines clinical medicine and rehabilitation medicine and has received a great deal of attention from the community, which makes it an iterative and updated research topic. This paper analyzes 23 years of relevant research in this field. Using the VOSview and CiteSpace software, trends in the field are reviewed, and a bibliometric-based analysis of core authors in the research field, highly productive and closely collaborating national institutions, core journals in the field, valuable cited references, and topical keywords in the field is conducted.

### 4.1. General information and global trends for stroke and quality of life

Based on data collected in the Web of Science core collection, between 2000 and 2022, 704 articles on stroke and quality of life were published by 3,261 authors from 1,271 institutions in 66 countries worldwide in 222 journals, citing 15,318 references from 4,211 journals. 18,922 times, with an increasing trend in the number of publications year by year. The rapid growth of published papers indicates that researchers are paying more attention to the quality of life after stroke. To our knowledge, this is the first article based on WoSCC and using bibliometric and visual analysis methods to assess stroke and quality of life, which may inform the direction of future research. Figure 4A shows the temporal distribution of publications in the field of quality of life after stroke, and although the number of publications declined considerably during 2015–2018, the annual number of papers increased dramatically from 2018 to 2021, reaching 77 papers and 2,236 citations in 2021 (Figure 4). Despite the overall decreasing trend in stroke mortality and morbidity worldwide, the socioeconomic burden of stroke survivors remains high and several countries are gradually starting to focus on the healthy quality of life of stroke survivors. The growing trend of publications indicates that this research area has received increasing attention from scholars in recent years, and we predict a great potential for growth in this research area.

### 4.2. Quality of global publications on stroke and quality of life

#### 4.2.1. Authors and co-cited authors

In this study, CiteSpace helped to identify the core authors with the highest number of publications and citations (Figures 5, 6), reflecting the authors’ contributions to the field and their collaborations. KIM S, who has the highest number of publications, works at Korea University College of Nursing and leads a team studying the relationship between Type D personality and quality of life in post-stroke patients. Type D personality has a high rate of stroke recurrence and high frequency of antidepressant medication use, and this study helps to reduce stroke recurrence, increase healthy behaviors in post-stroke patients and improve quality of survival (19). Donnan G and Anderson C, who have formed a partnership in the field of quality of health care, work at the National Stroke Institute at the University of Melbourne and Anderson C works at the University of Sydney, and their team has conducted observational studies of post-stroke patients using a follow-up approach to encourage stroke patients to participate in rehabilitation and receive medication and related care programs to improve HRQOL (20, 30); Author Chen C and author Wu C focused their research on rehabilitation therapies to improve patient function through common rehabilitation interventions, such as modern rehabilitation robotic assistive technology and traditional Chinese health care techniques (31–33). Author Kim S and author Kim J focus on longitudinal studies, as one of the important research methods in psychology, longitudinal study is a continuous observation of a population or individuals in that population to understand the dynamics of disease, nutritional health status or a health event over time. Cross-sectional associations cannot explain the relationship between post-stroke depression and quality of life, therefore Kim J et al. conducted a study on the longitudinal impact of depression on quality of life in stroke patients and found that post-stroke depression was associated with low quality of life in the acute phase of stroke and had a persistent negative impact on patients, emphasizing the need to assess patients for depression after the onset of stroke, in addition to medical interventions (34).

Similarly, we clustered and grouped the author co-citation networks using keywords, and the main research topics were Validity, neglect, Marathi, poststroke fatigue, and response shift, represented by William LS, Duncan PW, Carod-artal FJ, and The first cited article, Development of a Stroke-Specific Quality of Life Scale, was published 462 times in 1999 by Williams LS et al. Williams LS led the team that developed the SS-QoL, a patient-centered outcome measure designed to assess HRQOL specific to stroke patients. The SS-QoL is a patient-centered outcome measure designed to assess patient-specific HRQOL and has the advantage of being valid, reliable, and responsive, and is commonly used to assess the quality of life in stroke patients (11). More attention to the research results of core authors in this field will help to open our minds and promote research innovation.

#### 4.2.2. Countries and institutions

Combined with Table 7 it can be seen that the United States is the most published country in this field and is the central partner of other countries. The United States ranked first in the number and centrality of publications in quality of life after stroke research, with 104 publications and a centrality of 0.33. Stroke is the second leading cause of death worldwide, and as a developed country, stroke has also posed a major socioeconomic threat to the United States, so the United States has invested significant and research and medical resources to study stroke. Every year, the United States promotes awareness of the prevention and treatment of hypertension through various means. In addition, the United States, Australia, and the United Kingdom are the core research forces in the collaborative network and work closely with other countries.

**Table 7:** Top five countries in terms of count and centrality.

Rank	Country	Count	Country	Centrality
1	United States	104	United States	0.33
2	China	81	Australia	0.31
3	Australia	70	United Kingdom	0.17
4	South Korea	54	Nigeria	0.1
5	United Kingdom	46	Singapore	0.1

We used CiteSpace to cluster institutions and extracted cluster labels from the titles to obtain a total of seven clusters, the largest cluster was labeled long-term quality, and the main cited article was Early mobilization and quality of life after stroke findings from avert published in neurology in 2019, the University of Melbourne was the institution with the most citations in this cluster, and the University of Melbourne is both a high volume of publications and a high centrality (Table 8). The second largest cluster was labeled life scale, and the most cited institution was National Taiwan University, which was also the third most prolific institution; the third largest cluster was labeled kosco study, and the Jeonnam National University was the most cited institution in this cluster. In addition, Chinese institutions accounted for 81.6% of the top five publications (Table 8). According to the census, China’s population growth rate is much less than the prevalence of cerebrovascular disease, and as the world’s most populous and rapidly aging country, we speculate that China faces a great challenge in reducing stroke morbidity and mortality, and therefore institutions in China are investing more research in this research area resources and efforts.

**Table 8:** Top five institutions in terms of count and centrality.

Rank	Institution	Count	Institution	Centrality
1	Chinese University of Hong Kong	20	Deakin University	0.06
2	Chang Gung University	15	University of Melbourne	0.05
3	National Taiwan University	15	King’s College London	0.04
4	University of Melbourne	14	Austin and Repatriation Medical Centre	0.04
5	National Taiwan University Hospital	12	Chonnam National University	0.03

National and institutional collaboration helps researchers in this field to share resources and exchange knowledge and ideas, which is essential for the development of scientific research. Therefore, stronger collaborative networks should be established among more countries and institutions.

#### 4.2.3. Journals and co-cited journals

A total of 704 papers on stroke and quality of life were published in 223 journals. From the dual-map of journals (Figure 9), the cited journals were concentrated in neurology, sports, ophthalmology, medicine, medical, clinical, which are called frontiers of research. The cited journals are mainly in the fields of psychology, education, social, health, nursing, medicine, which are called knowledge base.

Table 2 shows that the top 10 journals account for 35% of the total, with the exception of Stroke, which has an IF = 10.17, and the remaining nine journals with low IFs, which we believe lack some groundbreaking discoveries and innovations in the field. Diseases (58.06) and Archives of physical medicine and rehabilitation (50.67), indicating that these three journals contain high quality articles and have received much attention in the field of stroke and quality of life. With 53 papers published over the past 23 years, Stroke is the most preferred journal for those in the field of stroke and quality of life research. As shown in Figure 1 and Table 3, the most frequently cited journal is Stroke, with 4,131 citations, and the first cited paper on quality of life in stroke survivors was also from Stroke, making Stroke arguably the most influential core journal in the field. In terms of centrality, an important journal in the field of stroke and quality of life research is Physical therapy (Table 3), which works closely with other journals. In addition, by combining Tables 2, 3, we found that four journals, Stroke, Disability and Rehabilitation, Quality of life research, and Archives of physical medicine and rehabilitation, are both important citation-giving and cited journals, and the articles in these journals reflect the underlying theories in this field of research. Future scholars should pay more attention to these journals in order to quickly access the latest international information and research advances in stroke and quality of life.

### 4.3. Research hotpot for stroke and quality of life.

#### 4.3.1. Co-cited references with the strongest citation bursts

A burst reference is a reference that has been cited frequently over a period of time, representing a considerable interest and concern of the researcher in a certain field. Therefore, analyzing the clues of burst references can effectively identify the research hotspots and research frontiers in the field (16). Table 9 lists the top 20 co-cited literature with the strongest citation bursts from 2000 to 2022. The light blue bar indicates that the reference has not yet appeared, the dark blue bar indicates that the reference has started to appear, and the red line represents the burst time.

**Table 9:** Top 20 references with the strongest citation bursts.

References	Year	Strength	Begin	End	2000–2022
Williams et al.	1999	6.83	2000	2004	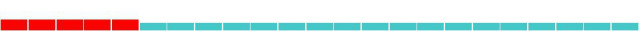
King et al.	1996	6.46	2000	2001	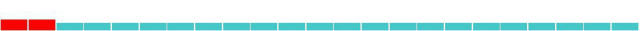
Carod-artal et al.	2000	6.5	2002	2005	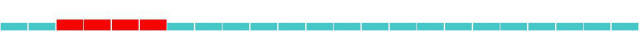
Kim et al.	1999	6.19	2002	2004	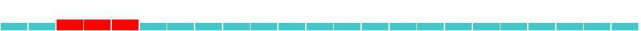
Sturm et al.	2004	**13.49**	2005	2009	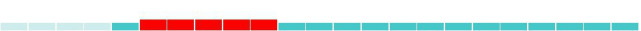
Hopman et al.	2003	6.48	2005	2008	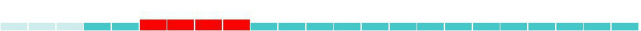
Jonsson et al.	2005	**11.5**	2006	2010	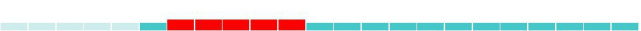
Nichols-larsen et al.	2005	5.96	2006	2010	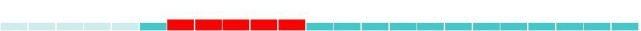
Paul et al.	2005	6.19	2007	2010	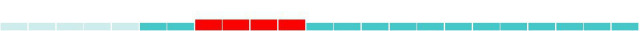
Haacke et al.	2006	7.46	2008	2011	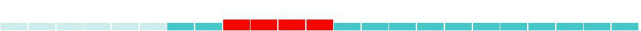
Kwok et al.	2006	6.96	2008	2011	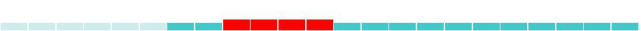
Patel et al.	2007	9.92	2009	2012	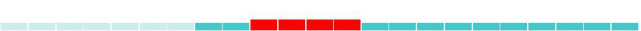
Muus et al.	2007	5.64	2009	2012	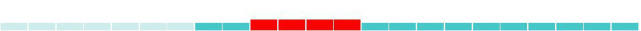
Carod-artal et al.	2009	**12.05**	2011	2014	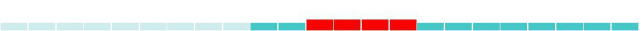
Dhamoon et al.	2010	5.94	2012	2015	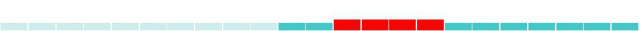
Abubakar et al.	2012	6.14	2014	2017	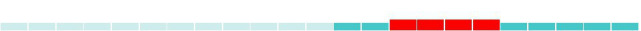
Bushnell et al.	2014	7.6	2015	2019	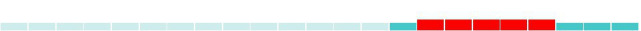
De Wit et al.	2016	6.38	2019	**2022**	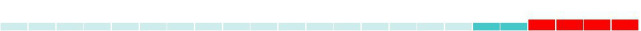
Ramos-lima et al.	2018	9.06	2020	**2022**	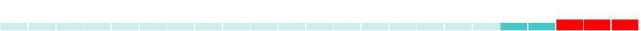
Kwon et al.	2018	5.7	2020	**2022**	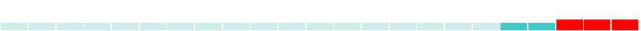

The bold values in table refer to the three co-cited references whose citation burst strength is greater than 10.

The first appearance of co-cited literature related to outbreaks was in 2000. The authors of the top three strongest sources of outbreaks were Sturm JW, Carod-artal FJ, and Jonsson AC. with the highest citation intensity (*n* = 13.49 citation bursts) coming from Sturm JW (35) and his team published in Stroke, who had previously studied disability data in community stroke survivors (36), and although disability is the most relevant patient clinical outcome, quality of life may be more relevant from the patient’s perspective (22). They assessed 2 years after stroke using Assessment of Quality of Life (AQOL) and most survivors had severely impaired HRQQL, and the determinants of HRQOL were disability, physical impairment, anxiety, and depression. Predictors were age, female, initial NIHSS score, neglect and low socioeconomic status. Carod-artal et al. (29) reviewed HRQOL assessment and determinants in stroke survivors. They described the need for HRQOL measurement tools in clinical practice and commonly used assessment scales. Many factors predict HRQOL in stroke survivors, with functional status and long-term disability being determinants, and psychosocial factors such as depression also affecting HRQOL and functional recovery in stroke survivors. In addition, they found that stroke caregivers who lacked family support and professional support also had generally lower HRQOL. Jonsson et al. (23) also found that caregivers may be under considerable stress and that caregivers have poorer quality of life than patients in terms of emotional and spiritual factors. The determinants of quality of life for caregivers are their own age and the functional status of the patient. Stroke research therefore needs to focus more on the overall functional capacity of survivors, comorbidities and psychosocial factors, and stroke rehabilitation needs to be survivor and caregiver oriented.

There are three articles with reference bursts ending in 2022, and these are commonly cited in recent years, which shows that the content and views in these references are hot spots that tend to be popular. de Wit et al. (37) used the EQ-VAS z-Norm score to compare the long-term HRQOL levels of stroke survivors with population normal levels and found that stroke had a 5-year stroke survivor’s HRQOL The effect of stroke on HRQOL was highly variable, with lower HRQOL scores associated with functional status, female, higher age (38), and lower socioeconomic status (39), but the study was limited by not considering psychosocial factors. Ramos-lima et al. (38) found that the most relevant predictor of QOL was functional status during assessment, and that patients with left hemisphere stroke usually have language impairment and worse HRQOL, so lifestyle changes and encouragement of physical activity are necessary. In addition, they found that orthotic use, although improving gait performance and controlling abnormal kinematics due to coordination deficits, also had a negative impact on HRQOL. Kwon et al. (40) evaluated the factors influencing HRQOL in stroke survivors and suggested that the development of rehabilitation programs must be tailored to the physical performance level of stroke survivors. Therefore, we need to pay attention to psychosocial factors that affect the health quality of life of survivors, and for post-stroke depression, the community should provide comprehensive medical care including depression screening and treatment. In addition, because it is difficult for stroke survivors to return to work and inadequate financial support limits medical care and participation in activities, which in turn exacerbates the lower quality of life of stroke survivors (41, 42), vocational rehabilitation should also receive attention.

#### 4.3.2. Keywords with the strongest citation bursts

Burst keywords are keywords that are frequently cited over a period of time and can reflect the cutting-edge topics in that research area. Table 10 shows the 20 most frequently cited keywords. The most cited keywords in the early stage include quality of life, stroke outcome, health status, and reliability, which shows that the quality of survival and scale reliability of patients after stroke are popular topics in the early stage of research in this field; The popular keywords in recent years include disease (2018–2022), care (2018–2022), global burden (2018–2022), and anxiety (2019–2022). The four research trends are as follows:

**Table 10:** Top 20 keywords with the strongest citation bursts.

Keywords	Year	Strength	Begin	End	2000–2022
Quality of life	2000	9.28	2000	2003	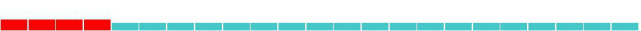
Stroke outcome	2000	6.43	2000	2010	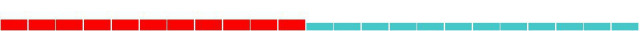
Health status	2000	5.36	2000	2007	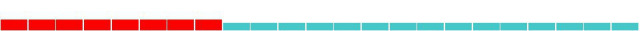
Reliability	2000	3.86	2000	2006	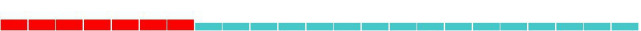
Health-related quality of life	2000	4.52	2003	2006	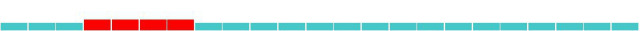
East Melbourne stroke	2000	6.62	2007	2013	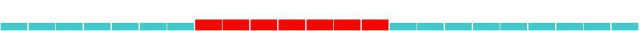
Follow up	2000	4.45	2008	2015	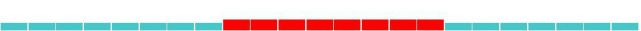
Determinant	2000	4.4	2008	2011	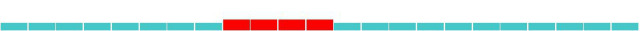
Induced movement therapy	2000	4.07	2009	2010	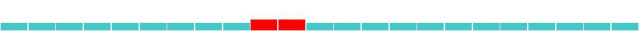
Randomized controlled trial	2000	4.07	2010	2016	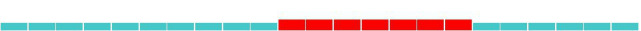
Community	2000	4	2013	2014	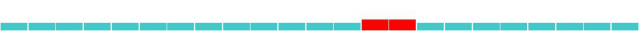
Poststroke	2000	4.32	2016	2022	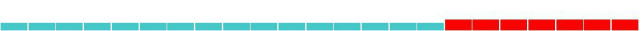
Therapy	2000	4.15	2016	2022	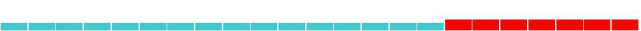
Trial	2000	3.98	2016	2020	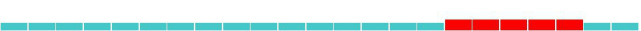
Risk factor	2000	4.55	2017	2019	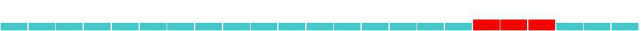
Disease	2000	4.7	2018	2022	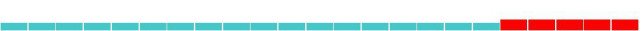
Care	2000	4.52	2018	2022	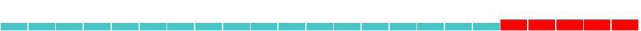
Global burden	2000	3.97	2018	2022	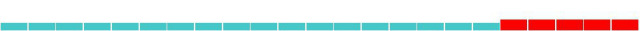
EQ-5D	2000	4.2	2019	2020	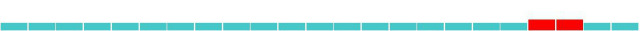
Anxiety	2000	4.13	2019	2022	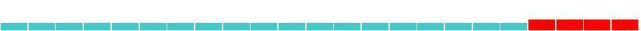

(1) Disease: Hypertension, diabetes mellitus, and obesity are considered risk factors for the occurrence of stroke (43), and the quality of survival of post-stroke patients includes depression in addition to these disease factors. suYeon Kwon et al. (40) suggested that rehabilitation interventions for post-stroke depression should be developed, and vocational rehabilitation and individualized physical activity should be developed to improve post-stroke patients’ HROQL (40).

(2) Care: Effective care after stroke has an important impact on improving the quality of patient survival. In the acute phase of stroke, the goal of care is to stabilize the patient’s condition through acute treatment. Rehabilitation, an important component of effective care, should be intervened as early as possible after the patient’s condition is stabilized. The goal of early rehabilitation is to encourage active participation of the patient, with support and education provided by caregivers and family members; the goal of late rehabilitation is to restore somatic function for social reintegration as much as possible (44).

(3) Global burden: The burden caused by stroke continues to increase globally (45). With approximately 2 million deaths due to stroke in China in 2017, stroke has become the leading cause of death. By documenting lifestyle, environmental and occupational exposures, and metabolic risk factors, it is possible to quantify the magnitude of the stroke burden associated with various risks and to identify prevention strategies at the global, regional, and national levels to reduce the social and economic burden of stroke (46, 47).

(4) Anxiety: There is a correlation between psychological factors and quality of life after stroke (48). The higher the level of depression and anxiety after stroke, the lower the patients’ HRQOL scores (37), and the development of rehabilitation programs is important to improve quality of life and reduce mortality, such as the development of qigong exercises as a complementary and alternative form of medicine that can reduce mental distress caused by stroke (32).

### 4.4. Significance

#### 4.4.1. Significance of published content to the study

Factors influencing QOL in stroke survivors can be categorized into demographic variables, associated vascular risk factors, neurological dysfunction, functional status, cognitive and behavioral factors, psychological factors, and social variables. Among these, the most relevant predictor of QOL is functional status. Therefore, programs tailored to the functional status of stroke survivors can help provide a better quality of life. In addition, the high prevalence of depression after stroke confirms the need for assessment and intervention for depression. The variables associated with depression are stay-at-home status, feminization, inadequate social support, rehabilitation, depression screening and community interventions for stroke survivors must take into account the support of other family members or caregivers, and the mental status of caregivers cannot be ignored. Therefore, post-stroke rehabilitation should be multifaceted, with mental health education along with improvement of physical function to improve the patient’s post-stroke quality of life.

#### 4.4.2. The practical significance of the study

This study uses data visualization to summarize and express complex information in the field of post-stroke quality of life research, which has important implications for hospitals, medical students, and the community engaged in stroke research. First, this study summarizes the current state of research and predictors of quality of life after stroke, emphasizing that psychosocial factors such as depression or anxiety cause the same or greater decline in quality of life than physical disability (49), suggesting that hospitals, universities and community should not only focus on the functional status of stroke patients, but should place a high priority on the psychological status of survivors and caregivers. This study can spread the knowledge of cerebrovascular diseases to the public and raise the awareness of cerebrovascular accident prevention, which is important to reduce the incidence of stroke. Secondly, this study summarized the important cooperative forces, classic literature, key literature and research hotspots in this field, and summarized the research progress and emerging research hotspots in the field of stroke and quality of life, which has great reference value for the scientific research of hospitals and universities engaged in stroke.

### 4.5. Strengths and limitations

To the best of our knowledge, this is the first article based on WoSCC and using bibliometric and visual analysis methods to assess 23 years of stroke and quality of life. This study includes 704 papers describing the current state of research on stroke and quality of life in terms of authors, countries, institutions, journals, references, and keywords, demonstrating the research base in the field, and also includes emergent references and emergent keywords that help to identify the research hotspots and future trends in the field.

However, this study also has some limitations. First, due to CiteSpace software and technical limitations, we only included relevant literature from the English database WoSCC and did not consider other databases; Second, this study chose English as inclusion criteria and sent out non-English articles, which may lead to research bias in published literature; Third, bibliometrics cannot fully consider the validity and scientific rigor of publications, and citation indicators may be influenced by the time of publication, and recently published studies may be underestimated, but we believe that our findings effectively represent global research in the field of post-stroke quality of life studies.

## 5. Conclusion

Through a visual analysis of stroke and quality of life research over the past 23 years, this study presents the current research findings and possible future research directions in stroke and quality of life. Kim S, Williams LS, Anderson C, and Ali M are important authors in this field, the United States, Australia and the United Kingdom are core research forces, and the Chinese University of Hong Kong in China has made the largest contribution in this field. The University of Melbourne is an important research institution, and stroke is the most influential core journal in the field. The main research interests are stroke disease progression and related complications, standardized post-stroke quality of life assessment measures and tools, care and rehabilitation of stroke survivors, and the global burden of disease. Topics related to post-stroke depression and anxiety, rehabilitation, care, and scales are emerging research hotspots. This analysis may enable academic collaboration and communication among countries, institutions, and scholars should be enhanced, and provides a useful basis for researchers to further understand the research hotspots, research priorities, and emerging trends in the field of stroke and quality of life.

## Author contributions

MC and YZ contributed significantly to analysis and manuscript preparation, performed the data analyses, and wrote the manuscript. LD helped perform the analysis with constructive discussions. XiG contributed to the conception of the study. All authors contributed to the article and approved the submitted version.

## Funding

This study was supported by Zhongshan Social Public Welfare and Basic Research Program (2021B1055).

## Conflict of interest

The authors declare that the research was conducted in the absence of any commercial or financial relationships that could be construed as a potential conflict of interest.

## Publisher’s note

All claims expressed in this article are solely those of the authors and do not necessarily represent those of their affiliated organizations, or those of the publisher, the editors and the reviewers. Any product that may be evaluated in this article, or claim that may be made by its manufacturer, is not guaranteed or endorsed by the publisher.
